# Masked Men at Guy's Hospital

**Published:** 1919-06-28

**Authors:** 


### MASKED MEN AT GUY'S HOSPITAL.

We are indebted to the *Evening Standard* for the following account of an interview with Mr. W. J. Curry, the Clerk to the Governors of Guy's Hospital, who was attacked in the counting-house on the afternoon of Friday, June 20:—

"They got me, but unfortunately I did not get them," said Mr. Curry.

"I was working at my desk in the counting-house on the ground floor," he continued, "when I was suddenly confronted by two armed and masked men, each having a pistol or revolver.

"I demanded, 'What do you want?' and came to close quarters with them.

"As I did so one of them was preparing a noose with a piece of rope he had in his hand.

"I tackled them, and tried to get through an awkward corner between them.

"While I was battling with one of them, the other was hammering at my head with an implement he had—some sort of fire-iron.

"As far as I remember they got me down on the floor in the hall on my way to the front square to get help. Both still had their masks on then, but I don't suppose they would run out with them on.

"I must have been stunned for a bit, and they bolted then through the front door into the quadrangle.

"But I regained my feet, and followed them as soon as possible, and shouted for help just at the entrance door.

"Then I saw them make their escape through the gate in the front railings.

"I don't remember any more until I found myself here, but I'm afraid they got away."

Mr. Curry, naturally, had no time to take particular notice of his assailants, but he states that as far as he could judge when they had masks on they were both young men. The masks were just pieces of black cloth, with eye-holes cut in.

The hero of this remarkable fight is a man of medium height but sturdy build, just over forty years of age. He has served in the Volunteers, but was claimed as indispensable during the war. One can imagine he would have been a pretty useful man in a patrol or bombing raid. His injuries are in the front, side, and back of the head, one being an open cut that has been stitched up.

The robbers had selected a good hour for the attack, but unfortunately they encountered the wrong man. Another miscalculation they made was with regard to the safe, which, even had they been successful, would not have yielded so big a haul as on some other days.

The first to come to Mr. Curry's aid was his lady clerk from upstairs, doctors and nurses were quickly on the scene, and the wounded man was rapidly attended to. When the police arrived they found in Mr. Curry's room the two masks, a broken steel shovel, and a long rope with a noose. The assailants had probably secreted themselves in some part of the building, and waited until it was closed, for Mr. Curry was working late at the time.

The plucky clerk has recovered to such an extent that he has been removed to his home.

